# On the Treatment of Wounds and Ulcers, and Hæmorrhage

**Published:** 1815-07

**Authors:** Richard Walker

**Affiliations:** of Oxford.


*For the Medical and Physical Journal.*

### *On the Treatment of Wounds and Ulcers, and Hæmorrhage*; by Mr. Richard Walker, of Oxford.

*(Continued from p. 468.)*

ON *Hæmorrhage.*—There are various means in use for stopping hæmorrhage; but the chief of these, and which are commonly resorted to when vessels of any magnitude are wounded, are by ligature or compression. The former of these methods, being the most secure, should be put in practice, in all cases which admit of it, in preference to compression.

In cases, however, where the ligature cannot be conveniently applied, as is not unfrequently the case, even in bloodblood-vessels of considerable magnitude, compression, dexterously applied, answers very effectually.

There are two modes of applying compression: the first is by plugging up the bleeding vessels and wound with agaric or lint, and then duly applying compress and bandage; the second method, and most modern one, is, instead of plugging up the wound and bleeding vessel with agaric or lint, to bring the lips of the wound in contact, and then using compression.

It is apparent, the latter method must be preferable, provided it be equally safe, because the progress towards healing by the first intention, as it is called, is going on at the same time.

Having myself, however, in a great many instances, witnessed the inconvenience and alarm arising from a second hæmorrhage, I prefer the former of the two methods, by compression, considering it by far as the most secure one; and, moreover, the delay in the cure, arising from the wound being plugged up, and somewhat dilated by the agaric, may be, in a great measure, obviated afterwards, by a judicious application of adhesive plaster.

The latter method may be adviseable in the instance of the wounded blood-vessels being situated upon or over a bone, as in the head, &c. where effectual compression, in this manner, may be made, and especially where the circumstances of obviating a scar is important.

After witnessing the effects of the different modes of stopping hæmorrhage, by compression, in an immense number of cases, by other persons, I finally determined upon the following method, differing, in several material respects, from that of either of the surgeons, and which I constantly use.

In the first place, I apply a sponge or cloth into the wound, so as to plug it up, and immediately apply the tourniquet, if the vessel be of such magnitude as to require this precaution. Secondly, after cleansing the wound, I apply small portions of agaric, chusing such as is of the most spongy texture, into the wound, compressing it strongly with my thumb, if the wound be small, but, if large, with a sponge which has been thoroughly wetted with cold water, and squeezed out, repeating this until the wound is completely filled; lastly, applying over the former, if the wound be small, one larger piece of agaric, as a pad or compress over the former, or several pieces, if the wound be so large as to require it; and over this, if requisite, a pad of lint, well floured. Thirdly, these are secured by adhesive plaster, in one strip, sufficiently broad, and of such a length, where the the part will admit of it, as to surround the limb entirely, and to admit of the ends wrapping over each other, upon the wound; and by way of further security, in cases which may seem to require it, a second plaster, in like manner over the former. Fourthly, a pad or folded compress of linen is placed over the wound, and the whole secured by a roller. If the plaster spread on cloth be properly adhesive, and duly applied, with respect to tightness, &c. the compress and bandage last mentioned are, in ordinary cases, an almost useless appendage, with respect to actual utility, though certainly never to be dispensed with. Lastly, the tourniquet might be continued loosely in its place, in order to be tightened, if there be occasion, and the part placed in an elevated situation. Care must be taken that the part be made as dry as possible, immediately before the adhesive plaster is applied.

Some time after the part may swell so much, and become so painful, as to render it necessary that the plaster, which will not give way, should be snipped round the edges sufficiently to give due room, without endangering fresh hæmorrhage; and the bandage loosened or clipped likewise, if it be necessary; which must be repeated, as occasion may require. It is scarcely necessary to state, that rest in bed, low diet, and coolness of the part, are indispensible, which latter may be effected, if necessary, by the application of cloths, soaked in cold water, and squeezed so dry as not to endanger the dressings being in any degree moistened, by which they might be loosened, and fresh hæmorrhage endangered.

The plaster should be occasionally clipped and even cut away, that part immediately covering the wound excepted, previously to dressing it, which should be deferred for several days, that is, until it is in some degree loosened or detached by the discharge.

At the first opening of the wound, it will be prudent to apply a piece of agaric immediately upon the part, with a little lint over, continuing the adhesive plaster in such a manner as to bring together, gradually, the separated lips of the wound, in the progress of healing, as nearly in contact as possible.*

* It may be proper to observe here, that the day or night before I intend opening the wound for the first time, I direct a poultice of bread and milk, or bread and water, to be applied, in order to loosen or detach the dressing from the part; and if any part of the dressing remain still so tight as to endanger fresh hæmorrhage by its removal, I suffer it still to remain, applying a piece of agaric or floured lint immediately upon the part, as a safe-guard, with the adhesive plaster and bandage as before.

The

The limb should be surrounded by a single strip of the adhesive plaster, as at first, in every succeeding dressing; and if economy be found requisite, in this or any other instance where adhesive plaster is used, the greater portion of the surrounding plaster may be suffered to remain, renewing that portion over the wound only, observing that each end of the latter wrap sufficiently over the other to keep the whole firm.

I had, some time since, a patient in whom the ulnar artery was divided, near the wrist, with whom I pursued the above method with such complete success, that he lost not a drop of blood after it was first dressed, nor suffered any considerable inconvenience, except being at first confined to his bed, during the cure, which was completed in about three weeks, and leaving but a very small scar.

It is of great importance to be able to judge where tying or passing a ligature round blood-vessels can be safely dispensed with, for various reasons, particularly because it is frequently necessary, for this purpose, to dilate or enlarge the wound, as in instances of punctures and small wounds, which, of course, occasions much pain, and protracts the cure.

I have mentioned the case above principally to show where ligatures may, by due management of other means, be ordinarily dispensed with, viz. in blood-vessels of less importance or magnitude than the above, where due pressure can be made; having occasionally witnessed cases of this description treated unnecessarily by ligature, and in some instances where much blood as well as time has been wasted in fruitless attempts to take up the vessel by ligature, which was at length effected by pressure alone.

Escharotics, as argenti nitras, cupri sulphas, &c. acting directly as styptics, without leaving a slough or eschar behind, as caustics do, are preferable, because, on the separation of the slough or eschar, the hæmorrhage returns. The application of lunar caustic is occasionally a useful styptic to a bleeding surface.

Cold is of essential service in cases of hæmorrhage, particularly in hot weather, and may be applied by cloth folded three or four times double, soaked in the coldest water, and then pressed nearly dry, laid over the dressings, and renewed occasionally. In summer, two or three pails full of water should be pumped off immediately before the water which is to be used. Care should still be taken that the part be placed and kept in a somewhat elevated position.

*Oxford.* RICHARD WALKER.

P.S.—Some practitioners are averse to the use of saturnine preparations preparations as an application, in consequence of the supposed deleterious effect which might ensue from its absorption into the system. I shall briefly observe, respecting this circumstance, that I have for many years witnessed the free use of such applications, upon extensive sores, and widely-extended abraded surfaces, and for a considerable length of time, without knowing an instance of any evil consequence arising from them; and, were there occasion, I should not hesitate to make use of them to myself.

Moreover, I might add the authority of an author and practitioner of great celebrity (see a System of Surgery, by Benj. Bell, 6th edit. vol. i. p. 38), who is decidedly of the same opinion. If the above prejudices against such applications are well founded, why have saturnine applications a place in our Pharmacopœias? I am well convinced that such applications possess a peculiar sedative astringent quality, for which there is no adequate substitute yet known.

It may not be superfluous to observe, that arsenical preparations are occasional applications, and without any consequent injury to the system.

In slight excoriations, or chafing as it is called, starch, or hair-powder, is a common and efficacious application.

It may be considered as a general rule, that unctuous applications, as cerates, &c. to a highly-inflamed or heated skin, especially if of a softish consistence, and in hot weather, are pernicious, and, if used at such time, should be constantly covered by cold damped cloths.

It should be recollected that the composition which forms the consistence of a cerate in winter, will become an unguent in summer.

In all instances of incised wounds, where there is no loss of substance, nor other natural impediment, the cicatrix formed or left after the cure should be a mere line, otherwise it may be considered that the surgeon has not effected so complete a cure as his art or profession admits of.

